# Interaction between body mass index and serum uric acid in relation to blood pressure in community-dwelling Japanese men

**DOI:** 10.1186/s40885-018-0087-3

**Published:** 2018-01-30

**Authors:** Ryuichi Kawamoto, Daisuke Ninomiya, Kensuke Senzaki, Teru Kumagi

**Affiliations:** 1 Department of Community Medicine, Ehime University Graduate School of Medicine, Toon-city, Ehime 791-0295 Japan; 2 Department of Internal Medicine, Seiyo Municipal Nomura Hospital, 9-53 Nomura, Nomura-cho, Seiyo-city, Ehime 797-1212 Japan

**Keywords:** Blood pressure, Uric acid, Body mass index, Interaction, Risk factor, Community-dwelling men

## Abstract

**Background:**

Few data is available on the association between body mass index (BMI), serum uric acid (SUA) levels and blood pressure (BP) categories in the disease continuum, when efforts for its prevention may be applicable.

**Methods:**

We performed a cross-sectional study to examine the association between BMI, SUA and BP in a community-dwelling sample of Japanese men. Individuals not on antihypertensive and uric acid lowering medications, and aged 50 to 90 years [817men aged 66 ± 9 (mean ± standard deviation) years] were recruited for the survey during a community based annual medical check-up. The main outcome was the presence of prehypertension [systolic BP (SBP) 120-139 mmHg and/or diastolic BP (DBP) 80-89 mmHg] and hypertension [SBP ≥ 140 and /or DBP ≥ 90].

**Results:**

In participants with a BMI of < 21.0 kg/m^2^, increased SUA levels were positively associated with SBP and DBP, but in those with a BMI of ≥ 21.0 kg/m^2^, increased SUA levels were negatively associated with SBP and DBP. The interaction between BMI and SUA as well as BMI and SUA was a significant and independent determinant for both SBP (β = − 1.125, *p* = 0.001) and DBP (β = − 0.995, *p* = 0.005). Among participants, the respective prevalence of normotension, prehypertension, and hypertension was 19.5% and 53.7%, and 19.8%. The prevalence of normotension and prehypertension decreased with increasing BMI and the prevalence of hypertension increased with increasing BMI. In participants with a BMI ≥ 21.0 kg/m^2^, the adjusted-odds ratio of SUA for hypertension was 0.75 (95% CI, 0.59-0.95) compared with normotension and 0.82 (0.70-0.96) compared with prehypertension. In those with a BMI of < 21.0 kg/m^2^, these associations were not shown.

**Conclusion:**

BMI may modify the association between SUA and blood pressure status among community-dwelling men.

## Background

Hypertension is likely the most common disease in Japan and is strongly associated with an increased risk of cardiovascular disease (CVD). The Seventh Report of the Joint National Committee on Prevention, Detection, Evaluation and Treatment of High Blood Pressure (JNC 7) defined a systolic blood pressure (SBP) of 120 to 139 mmHg and/or diastolic blood pressure (DBP) 80 to 89 mmHg as prehypertension [1] based on the evidence of a modestly increasing risk of CVD among individuals with such levels [2]. Many studies demonstrated that the prehypertensive group had a higher body mass index (BMI), central obesity, a family history of hypertension, a sedentary lifestyle, eating high sodium foods, smoking, excessive alcohol intake, impaired glucose tolerance/diabetes, higher levels of blood glucose, low-density lipoprotein cholesterol (LDL-C), and triglycerides (TG), and lower levels of high-density lipoprotein cholesterol (HDL-C) than the normotensive group [3–6]. The Jichi Medical School Cohort Study which enrolled 4706 males and 7342 females from among the general Japanese population suggested that BMI > 23.0 kg/m^2^ was the strongest determinant of prehypertension [7]. Thus lifestyle modification or even medical treatment is recommended for individuals with prehypertension [8].

Serum uric acid (SUA) is the end product of endogenous and dietary purine metabolism in humans, and is catalyzed by the enzyme xanthine oxidase, which is involved in producing reactive-oxygen species (ROS). Its excess accumulation can lead to various diseases [9]. SUA is closely associated with an increased risk of prehypertension [10, 11], hypertension [12], metabolic syndrome [13], and cardiovascular disease (CVD) [14, 15]. However, despite an association between serum SUA level and these conditions, SUA may not be considered a risk in these conditions [16], but rather as biologically inert or possibly anti-inflammatory because it can function as an antioxidant [17]. Increased SUA was significantly elevated in a linear manner. Moreover, increased BMI and weight loss may represent an effective nonmedical strategy for reducing SUA levels, especially in postmenopausal women and men [18]. Thus, the relationships between SUA and BP of participants categorized by BMI level may be different, and an interactive effect between BMI and SUA on blood pressure may be considered.

The aim of this study was to evaluate the prevalence of prehypertension and hypertension, and its association with BMI, SUA and other confounding factors {e.g., age, habits, lipid, fasting plasma glucose (FPG), renal function, and liver function} using cross-sectional data from community-dwelling Japanese men aged of **≥**50 years.

## Methods

### Study population

**Fig. 1:**
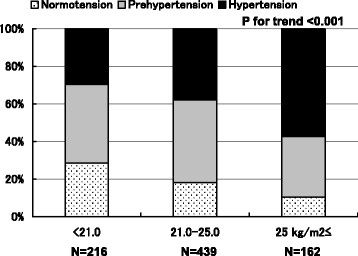
Prevalence of blood pressure status of participants categorized by body mass index. The prevalence of normotension and prehypertension decreased with increasing BMI and the prevalence of hypertension increased with increasing BMI (*p* < 0.001). *P*-value;χ^2^ test

The present study was designed as a part of the Nomura study [19]. Participants were selected through a community-based annual check-up process in a rural town located in Ehime prefecture, Japan. Baseline clinical characteristics including anthropometric parameters were obtained from the subject’s personal health records of the evaluated medical check-up. Other characteristics such as smoking and alcohol habit, medication, and history of CVD were investigated by individual interviews using a structured questionnaire. After excluding individuals with antihypertensive and UA lowering medications, the final study sample included only eligible persons (Fig. 1). This study was approved by the ethics committee of Ehime University School of Medicine, and written informed consent was obtained from each subject.

### Evaluation of risk factors

Information on demographic characteristics and risk factors was collected using clinical files. BMI was calculated by dividing weight (in kilograms) by the square of height (in meters). Smoking status was defined as the number of cigarette packs per day multiplied by the number of years smoked (pack year), and the participants were classified into never smokers, past smokers, light smokers (< 30 pack・year) and heavy smokers (≥30 pack・year). Daily alcohol consumption was measured using the Japanese liquor unit in which a unit corresponds to 22.9 g of ethanol, and the participants were classified into never drinkers, occasional drinkers (< 1 unit/day), light drinkers (< 2 unit/day), and heavy drinkers (≥2 unit/day). We measured SBP and DBP in the right upper arm of participants in the sedentary position using an automatic oscillometric blood pressure recorder while the participants were seated after having rested for at least 5 min. Appropriate cuff bladder size was determined at each visit based on arm circumference. Normotension was defined as not being on antihypertensive medication and having a SBP < 120 mmHg and DBP < 80 mmHg. Prehypertension was defined as not being on antihypertensive medication and having a SBP of 120 to 139 mmHg and/or DBP of 80 to 89 mmHg. Hypertension was defined as SBP ≥140 mmHg and/or DBP ≥90 mmHg (9). Total cholesterol (T-C), TG, HDL-C, FPG, creatinine (enzymatic method), SUA, aspartate transaminase (ALT), and γ-glutamyl transpeptidase (GGT) were measured during fasting. LDL-C levels were calculated by the Friedewald formula (14). Participants with TG levels ≥400 mg/dL were excluded. The presence of diabetes and CVD was defined as a history of treatment for diabetes and CVD. Estimated glomerular filtration ratio (eGFR) was calculated using CKD-EPI equations modified by a Japanese coefficient: Male, Cr ≤0.9 mg/dl, 141 × (Cr/0.9) ^–0.411^ × 0.993 ^age^ × 0.813; Cr > 0.9 mg/dl, 141 × (Cr/0.9) ^–1.209^ × 0.993 ^age^ × 0.813 [20].

### Statistical analysis

Statistical analysis was performed using IBM SPSS Statistics Version 20 (Statistical Package for Social Science Japan, Inc., Tokyo, Japan). All values are expressed as mean ± standard deviation (SD), unless otherwise specified. Data for TG, FPG, ALT, and GGT were skewed, and are presented as median (interquartile range) values, and were log-transformed for analysis. Differences in means and prevalence among the groups were analyzed by Student’s t-test for continuous data and χ^2^ test for categorical data. Correlations between various characteristics and BP were determined using age-adjusted partial Pearson’s correlation test and multiple linear regression analysis. Logistic regression analyses were used to test significant factors of prehypertension and hypertension (versus normotension), with prehypertension and hypertension serving as the dichotomous outcome variables, and age, BMI, alcohol consumption, smoking status, history of CVD, lipids, antilipidemic medication, FPG, antidiabetic medication, SUA, eGFR, ALT, and GGT as the confounding factors. The synergistic effect of BMI and SUA levels on blood pressure was evaluated using a general linear model. A value of *p* < 0.05 was considered significant.

## Results

The participants comprised 817 men aged 66 ± 9 years (range, 50-90) who do not take antihypertensive and UA lowering medications. Mean BMI in the study sample was 22.9 kg/m^2^ (SD, 2.8), with 26.4% being underweight (BMI < 21.0 kg/m^2^), 53.7% normal weight (BMI, 21.0 to 24.9 kg/m^2^), 19.8% overweight or obese (BMI ≥25 kg/m^2^). Table 1 shows the background characteristics of participants categorized by BMI. BMI, SBP, DBP, TG, LDL-C, prevalence of antilipidemic medication, FPG, SUA, ALT, and GGT were significantly high in the high BMI group, but HDL-C was low in the low BMI group. There were no inter-group differences regarding prevalence of alcohol consumption, smoking status, history of CVD, prevalence of antidiabetic medication, and eGFR.

**Table 1:** Characteristics of participants categorized by body mass index

	Body mass index category (kg/m^2^)			
Characteristics *N* = 817	< 21.0	21.0-24.9	≥25.0	*P*-value
	*N* = 216	*N* = 439	*N* = 162	
Age (years)	67 ± 9	67 ± 9	63 ± 9	**< 0.001**
Body mass index (kg/m^2^)	19.5 ± 1.1	23.0 ± 1.1	27.0 ± 2.0	**< 0.001**
Alcohol consumption^a^ (%)	37.0/20.8/9.3/32.9	42.6/21.9/9.1/26.4	34.6/30.2/7.4/27.8	0.157
Smoking status^b^ (%)	25.5/30.6/25.9/18.1	25.1/27.6/26.4/21.0	23.5/28.4/24.7/23.5	0.910
History of CVD, N (%)	16 (7.4)	41 (9.3)	11 (6.8)	0.514
Systolic blood pressure (mmHg)	132 ± 21	136 ± 19	142 ± 18	**< 0.001**
Diastolic blood pressure (mmHg)	79 ± 11	82 ± 10	85 ± 11	**< 0.001**
Triglycerides (mg/dL)	79 (59-101)	95 (72-141)	119 (81-161)	**< 0.001**
HDL cholesterol (mg/dL)	67 ± 17	58 ± 15	53 ± 13	**< 0.001**
LDL cholesterol (mg/dL)	103 ± 28	114 ± 30	115 ± 33	**< 0.001**
Antilipidemic medication, N (%)	3 (1.4)	25 (5.7)	9 (5.6)	**0.035**
Fasting plasma glucose (mg/dL)	98 (91-114)	102 (93-117)	103 (94-120)	**0.042**
Antiidiabetic medication, N (%)	6 (2.8)	28 (6.4)	8 (4.9)	0.145
Serum uric acid (mg/dL)	5.3 ± 1.2	5.7 ± 1.3	5.9 ± 1.2	**< 0.001**
eGFR (ml/min./1.73m^2^)	79.8 ± 13.9	77.0 ± 14.3	78.6 ± 15.3	0.051
Aspartate transaminase (IU/L)	17 (14-23)	19 (15-25)	23 (17-32)	**< 0.001**
γ-glutamyl transpeptidase (IU/L)	29 (20-43)	30 (22-49)	44 (28-69)	**< 0.001**

Data for triglycerides, fasting plasma glucose, aspartate transaminase, and γ-glutamyl transpeptidase were skewed and are presented as median (interquartile range) values, and were log-transformed for analysis. *P*-value: Student’s t-test for continuous variables or the χ^2^ -test for categorical variables. Bolded numbers indicate significance

*CVD* cardiovascular disease, *HDL* high-density lipoprotein, *LDL* low-density lipoprotein, *eGFR* estimated glomerular filtration rate. Data presented are mean ± standard deviation

^a^Daily alcohol consumption was measured using the Japanese liquor unit in which a unit corresponds to 22.9 g of ethanol, and the participants were classified into never, occasional, light daily (< 2 unit/day), and heavy daily drinkers (≥ 2 unit/day)

^b^Smoking status [never-smoker, past-smoker, light smoker (< 30 pack · year), and heavy smoker (≥ 30 pack · year)]

Among them, the respective prevalence of normotension, prehypertension, and hypertension was 19.5% and 53.7%, and 19.8%. The prevalence of normotension and prehypertension decreased with increasing BMI and the prevalence of hypertension increased with increasing BMI (Fig. 1).

**Fig. 2:**
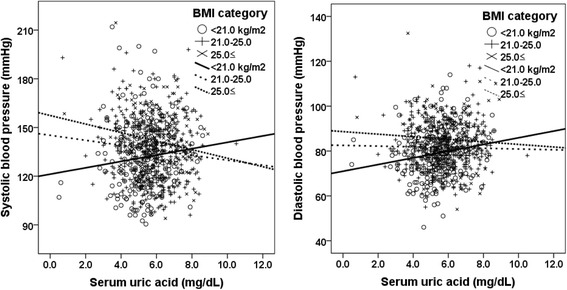
Correlation between serum uric acid and blood pressure status of participants categorized by body mass index. In body mass index (BMI) < 21.0 kg/m^2^, serum uric acid correlated positively with both systolic blood pressure (SBP) and diastolic blood pressure (DBP) (*r* = 0.112, *p* = 0.100 and *r* = 0.163, *p* = 0.016, respectively), but in BMI ≥21.0 kg/m^2^ serum uric acid correlated negatively with both SBP and DBP (BMI 21-25 kg/m^2^, *r* = − 0.108, *p* = 0.024 and *r* = − 0.022, *p* = 0.651; BMI ≥25.0 kg/m^2^, *r* = − 0.178, *p* = 0.023 and *r* = − 0.064, *p* = 0.421, respectively). Analysis of covariance showed that three regression lines in each graph were significantly different (SBP, F = 8.139, *P* = 0.004 and DBP, F = 5.199, *P* = 0.023, respectively)

In addition to their direct associations, we observed a synergistic effect between BMI category and SUA levels on BP status in Fig. 2. In BMI < 21.0 kg/m^2^, SUA correlated positively with both SBP and DBP (*r* = 0.112, *p* = 0.100 and *r* = 0.163, *p* = 0.016, respectively), but in BMI ≥25.0 kg/m^2^, SUA correlated negatively with both SBP and DBP (*r* = − 0.178, *p* = 0.023 and *r* = − 0.064, *p* = 0.421, respectively). Analysis of covariance showed that three regression lines in each graph were significantly different (SBP, F = 8.139, *p* = 0.004 and DBP, F = 5.199, *p* = 0.023, respectively).

Table 2 shows the background characteristics of participants categorized by BMI (i.e., < 21.0 kg/m^2^ and ≥21.0 kg/m^2^) and blood pressure status. In hypertensive group with a BMI < 21.0 kg/m^2^, prevalence of smoking status and GGT were significantly higher than normotensive group. In hypertensive group with a BMI ≥21.0 kg/m^2^, age and BMI as well as smoking status and GGT were also significantly higher, but SUA was significantly lower than normotensive group.

**Table 2:** Characteristics of participants categorized by body mass index and blood pressure status

	Body mass index < 21.0 kg/m^2^ *N* = 216				Body mass index ≥21.0 kg/m^2^ *N* = 601			
Characteristics	Normotension	Prehypertension	Hypertension	*P*-value	Normotension	Prehypertension	Hypertension	*P*-value
*N* = 817	*N* = 62	*N* = 90	*N* = 64		*N* = 97	*N* = 245	*N* = 259	
Age (years)	65 ± 10	69 ± 9	68 ± 8	0.083	63 ± 8	67 ± 9	66 ± 9	**0.002**
Body mass index (kg/m^2^)	19.4 ± 1.0	19.6 ± 1.1	19.6 ± 1.1	0.341	23.6 ± 2.1	23.7 ± 2.1	24.5 ± 2.4	**< 0.001**
Alcohol consumption (%)	40.3/21.0/11.2/27.4	34.4/27.8/10.0/27.8	37.5/10.9/6.3/45.3	0.099	43.3/19.6/10.3/26.8	37.1/27.3/8.6/26.9	42.5/22.8/8.1/26.6	0.731
Smoking status (%)	30.6/37.1/21.0/11.3	26.7/28.9/32.2/12.2	18.8/26.6/21.9/32.8	**0.011**	22.7/30.9/25.8/20.6	30.6/30.2/22.4/16.7	19.7/24.3/29.3/26.6	**0.012**
History of CVD, N (%)	3 (4.8)	10 (11.1)	3 (4.7)	0.214	6 (6.2)	28 (11.4)	18 (6.9)	0.130
Triglycerides (mg/dL)	79 (57-97)	79 (63-100)	80 (62-108)	0.266	110 (74-151)	99 (74-144)	100 (72-146)	0.997
HDL cholesterol (mg/dL)	68 ± 17	64 ± 16	70 ± 18	0.110	55 ± 16	57 ± 15	58 ± 14	0.278
LDL cholesterol (mg/dL)	101 ± 27	102 ± 28	108 ± 31	0.327	118 ± 29	114 ± 30	114 ± 33	0.396
Antilipidemic medication, N (%)	0	2 (2.2)	1 (1.6)	0.511	6 (6.2)	17 (6.9)	11 (4.2)	0.413
Fasting plasma glucose (mg/dL)	95 (89-111)	97 (89-112)	102 (94-120)	0.213	102 (92-116)	103 (91-117)	102 (94-120)	0.141
Antiidiabetic medication, N (%)	4 (6.5)	0	2 (3.1)	0.058	3 (3.1)	18 (7.3)	15 (5.8)	0.322
Serum uric acid (mg/dL)	5.1 ± 1.1	5.4 ± 1.3	5.6 ± 1.2	0.059	6.0 ± 1.1	5.8 ± 1.3	5.6 ± 1.3	**0.044**
eGFR (ml/min./1.73m^2^)	81.0 ± 14.8	78.3 ± 12.9	80.9 ± 14.4	0.372	76.8 ± 13.4	76.4 ± 14.5	78.5 ± 15.1	0.252
Aspartate transaminase (IU/L)	19 (15-24)	17 (13-22)	18 (14-24)	0.102	18 (15-24)	20 (15-26)	20 (16-27)	0.084
γ-glutamyl transpeptidase (IU/L)	27 (20-37)	27 (19-38)	35 (21-64)	**0.004**	29 (21-43)	34 (22-50)	36 (25-65)	**< 0.001**

Data for triglycerides, fasting plasma glucose, aspartate transaminase, and γ-glutamyl transpeptidase were skewed and were log-transformed for analysis. *P*-value: Student’s t-test for continuous variables or the χ^2^ -test for categorical variables. Bolded numbers indicate significance

Table 3 shows the relationship between various characteristics and blood pressure status of participants categorized by BMI. Age-adjusted partial Pearson’s correlation coefficient showed that SUA correlated positively with both SBP and DBP in participants with a BMI < 21.0 kg/m^2^ but correlated negatively with SBP in participants with a BMI ≥21.0 kg/m^2^.

**Table 3:** Age-adjusted relationship between various characteristics and blood pressure status of participants categorized by body mass index

	Body mass index < 21.0 kg/m^2^ *N* = 216		Body mass index ≥21.0 kg/m^2^ *N* = 601	
	Systolic blood pressure	Diastolic blood pressure	Systolic blood pressure	Diastolic blood presure
Characteristics *N* = 817	Partial r (*P*-value)	Partial r (*P*-value)	Partial r (*P*-value)	Partial r (*P*-value)
Body mass index	0.105 (0.127)	0.093 (0.174)	**0.190 (< 0.001)**	**0.153 (< 0.001)**
Alcohol consumption	**0.137 (0.044)**	0.107 (0.119)	−0.008 (0.844)	−0.030 (0.469)
Smoking status	**0.205 (0.003)**	**0.245 (< 0.001)**	**0.142 (< 0.001)**	**0.156 (< 0.001)**
History of CVD (Yes = 1, No = 0)	−0.061 (0.371)	−0.020 (0.766)	− 0.051 (0.216)	− 0.051 (0.209)
Triglycerides	0.047 (0.489)	0.112 (0.100)	0.031 (0.446)	0.064 (0.116)
HDL cholesterol	0.078 (0.255)	0.081 (0.240)	0.054 (0.186)	0.061 (0.138)
LDL cholesterol	−0.027 (0.689)	0.057 (0.404)	−0.019 (0.643)	0.000 (0.994)
Antilipidemic medication (Yes = 1, No = 0)	0.068 (0.318)	0.018 (0.791)	**−0.098 (0.017)**	−0.040 (0.334)
Fasting plasma glucose	**0.138 (0.043)**	0.059 (0.392)	**0.113 (0.005)**	0.029 (0.476)
Antiidiabetic medication (Yes = 1, No = 0)	−0.056 (0.410)	−0.064 (0.349)	0.007 (0.871)	−0.051 (0.214)
Serum uric acid	**0.134 (0.049)**	**0.153 (0.025)**	**−0.095 (0.020)**	−0.034 (0.401)
Estimated GFR	**0.184 (0.007)**	0.083 (0.223)	**0.083 (0.041)**	0.015 (0.719)
Aspartate transaminase	0.001 (0.983)	0.007 (0.917)	**0.084 (0.040)**	0.080 (0.051)
γ-glutamyl transpeptidase	**0.184 (0.007)**	**0.240 (< 0.001)**	**0.171 (< 0.001)**	**0.202 (< 0.001)**

r, Pearson’s partial correlation coefficients adjusted for age. Data for triglycerides, fasting plasma glucose, aspartate transaminase, and γ-glutamyl transpeptidase were skewed and were log-transformed for analysis. Bolded numbers indicate significance

Table 4 shows multivariate-adjusted relationship between various characteristics and blood pressure status of participants categorized by BMI. Multiple linear regression analysis showed that SUA was significantly and positively associated with both SBP and DBP in participants with a BMI < 21.0 kg/m^2^, but negatively associated with both SBP and DBP in participants with a BMI ≥21.0 kg/m^2^, independently of other confounding factors.

**Table 4:** Multivariate-adjusted relationship between various characteristics and blood pressure status of participants categorized by body mass index

	Body mass index category < 21.0 kg/m^2^ *N* = 216		Body mass index category ≥ 21.0 kg/m^2^ *N* = 601	
	Systolic blood pressure	Diastolic blood pressure	Systolic blood pressure	Diastolic blood pressure
Characteristics *N* = 817	β(P-value)§	β(P-value)§	β(P-value)§	β(P-value)§
Age	**0.375 (< 0.001)**	0.065 (0.396)	**0.275 (< 0.001)**	−0.045 (0.332)
Body mass index	–	–	**0.175 (< 0.001)**	**0.141 (0.001)**
Alcohol consumption	0.124 (0.064)	–	–	−0.046 (0.247)
Smoking status	**0.175 (0.010)**	**0.182 (0.014)**	**0.114 (0.006)**	**0.102 (0.020)**
History of CVD (Yes = 1, No = 0)	–	–	–	−0.042 (0.307)
Triglycerides	–	–	–	0.064 (0.167)
HDL cholesterol	–	–	–	0.083 (0.067)
LDL cholesterol	–	**0.162 (0.018)**	–	–
Antilipidemic medication (Yes = 1, No = 0)	–	–	**− 0.090 (0.020)**	–
Fasting plasma glucose	**0.151 (0.020)**	0.087 (0.197)	**0.088 (0.024)**	–
Antiidiabetic medication (Yes = 1, No = 0)	–	− 0.060 (0.370)	–	−0.041 (0.302)
Serum uric acid	**0.178 (0.011)**	**0.155 (0.028)**	**−0.127 (0.001)**	**−0.102 (0.019)**
Estimated GFR	**0.272 (< 0.001)**	**0.170 (0.030)**	–	− 0.036 (0.429)
Aspartate transaminase	–	− 0.086 (0.243)	–	–
γ-glutamyl transpeptidase	–	**0.243 (0.002)**	**0.124 (0.007)**	**0.157 (0.001)**
R^2^	**0.167 (< 0.001)**	**0.163 (< 0.001)**	**0.127 (< 0.001)**	**0.084 (< 0.001)**

Only factors remained in the final model were shown. Data for triglycerides, fasting plasma glucose, aspartate transaminase, and γ-glutamyl transpeptidase were skewed and were log-transformed for analysis. Bolded numbers indicate significance

*β* standard coefficient, *R*^*2*^ coefficient of determination

§Multivariate adjusted for all confounding factors in Table 1 by multiple linear regression analysis using backward elimination method

We assessed the statistical significance of the synergistic relationship using a general linear model with the following confounding factors: age, BMI, alcohol consumption, smoking status, history of CVD, TG, HDL-C, LDL-C, prevalence of antilipidemic medication, FPG, prevalence of antidiabetic medication, SUA, eGFR, ALT, GGT, and the interaction between BMI and SUA (Table 5). The interaction between increased BMI and SUA as well as BMI and SUA were significant and independent determinants for SBP (β = − 1.125, *p* = 0.001) and DBP (β = − 0.995, *P* = 0.005), independently of other confounding factors.

**Table 5:** Interaction between body mass index and uric acid on blood pressure status

	Systolic blood pressure	Diastolic blood pressure
Characteristics *N* = 817	β(*P*-value)§	β(P-value)§
Age	**0.294 (< 0.001)**	–
Body mass index	**0.715 (< 0.001)**	**0.620 (< 0.001)**
Smoking status	**0.117 ((0.001)**	**0.137 (< 0.001)**
LDL cholesterol	–	0.057 (0.096)
Fasting plasma glucose	**0.092 (0.006)**	–
Serum uric acid	**0.881 (0.002)**	**0.776 (0.008)**
Estimated GFR	**0.092 (0.016)**	–
γ-glutamyl transpeptidase	**0.119 (0.002)**	**0.178 (< 0.001)**
Body mass index^a^ serum uric acid	−**1.125 (0.001)**	**−0.995 (0.005)**
R^2^	**0.148 (< 0.001)**	**0.118 (0 < 0.001)**

Data for fasting plasma glucose, triglycerides, aspartate transaminase, and γ-glutamyl transpeptidase were skewed and were log-transformed for analysis. Bolded numbers indicate significance

*β* standard coefficient, *R*^*2*^ coefficient of determination

§Multivariate adjusted for all confounding factors in Table 1 by multiple linear regression analysis using backward elimination method. Only factors remained in the final model were shown

^a^Interaction between body mass index and serum uric acid

Table 6 shows the odds ratios (ORs) {95% confidence interval (CI)} of SUA for blood pressure status of participants categorized by BMI. In participants with a BMI ≥21.0 kg/m^2^, the odds ratio of SUA for hypertension was 0.75 (95% CI, 0.59-0.95) compared with normotension, and 0.82 (0.70-0.96) for hypertension compared with prehypertension. In subjects with a BMI < 21.0 kg/m^2^, these associations were not shown.

**Table 6:** Association between serum uric acid levels and blood pressure status of participants categorized by body mass index

Characteristic *N* = 817	Body mass index < 21.0 kg/m^2^			Body mass index ≥21.0 kg/m^2^			*P*-interaction
	*N* = 216	OR (95% CI)	*P-*value §	*N* = 601	OR (95% CI)	*P-*value §	
Prehypertension VS Normotension							
Serum uric acid	90/62	1.25 (0.91-1.72)	0.168	245/97	0.95 (0.77-1.18)	0.650	0.073
Hypertension VS Normotension							
Serum uric acid	64/62	1.53 (0.99-2.38)	0.058	259/97	**0.75 (0.59-0.95)**	**0.018**	**0.004**
Hypertension VS Prehypertension							
Serum uric acid	64/90	1.26 (0.92-1.74)	0.149	259/245	**0.82 (0.70-0.96)**	**0.012**	0.077

Data for fasting plasma glucose and γ-glutamyl transpeptidase were skewed and log-transformed for analysis. Bolded numbers indicate significance

*OR* odds ratio, *CI* confidence interval, VS versus

§Multivariate adjusted for age, body mass index, smoking status, LDL cholesterol, prevalence of antilipidemic medication, fasting plasma glucose, Estimated GFR, and γ-glutamyl transpeptidase, which were significant in Table 4

## Discussion

In this cross-sectional, population-based study of 817 middle-aged and elderly men, we set out to determine the prevalence of prehypertension and hypertension, as defined by the JNC-7 criteria [1], and its association with BMI and SUA. This study showed that increased SUA levels were positively associated with SBP and DBP in participants with a BMI < 21.0 kg/m^2^, but negatively in those with a BMI ≥21.0 kg/m^2^. The effect of significant interaction between BMI and SUA on blood pressure indicated that increased SUA was a risk factor for elevated blood pressure in underweight participants, but was not a predictor among normal- or overweight participants. To our knowledge, few studies have indicated that BMI may modify the association between SUA and blood pressure status.

In men aged < 40 years, SUA was significantly associated with SBP (β = 0.25, *p* = 0.002) and DBP (β = 0.41, *p* < 0.001) after adjustment for age, diabetes, dyslipidemia, BMI, and eGFR, but the association was not significant in those ≥60 years [21]. From a meta-analysis of 25 studies with 97,824 participants assessing the association between UA and incident hypertension, it was suggested that hyperuricemia may modestly increase the risk of hypertension incidence [22]. From a meta-analysis of 18 prospective cohort studies representing data from 55,607 participants, it was shown that hyperuricemia is associated with an increased risk for incident hypertension, independent of traditional hypertension risk factors. This risk appears to be more pronounced in younger individuals and women [12]. Thus, one can suggest that hyperuricemia-related pathogenetic mechanisms may be more dominant in earlier stages of hypertension than later stages when salt-sensitivity becomes apparent [12]. Our study findings indicate that we should consider the effect of BMI as well as age on the relationship between SUA and blood pressure.

The mechanism whereby BMI may modify the association between SUA and blood pressure status are not completely understood. SUA induces endothelial cell dysfunction via nitric oxide (NO) synthetase [23] and directly involves stimulation of the renin-angiotensin system [24]. SUA alters the proliferation/migration of endothelial and vascular smooth muscle cells [25]. These findings may provide insight into a pathogenic mechanism by which UA may induce hypertension and vascular disease [26]. A recent rodent model with induced hyperuricemia demonstrated that UA might have a pathogenic role in the development of renal afferent arteriolopathy and tubulointerstitial disease, leading to hypertension [27]. The renal lesions and the development of hypertension were prevented by lowering UA levels with allopurinol or benziodarone, which inhibits xanthine oxidase and hence blocks both UA and oxidant formation, which are reversed by angiotensin-converting enzyme inhibition [28].

Moreover, SUA also reflects systemic inflammation [29], oxidative stress [30] and is more strongly associated with insulin resistance [31, 32] and other cardiovascular risk factors such as BMI, BP, T-C, HDL-C, TG, and FPG [31, 33]. Increased BMI is also significantly associated with various CVD risk factors. These risk factors cause endothelial dysfunction [34], the loss of vasomotor reactivity [35] and arterial stiffness [36]. Such pathophysiology induced by increased BMI may be greater than that of SUA. Thus, SUA could not be an independent risk factor for increased SBP in participants with a BMI ≥21.0 kg/m^2^. We suggest that SUA may be more important in participants with a BMI < 21.0 kg. It has been demonstrated that increased SUA induces increased BP that is initially reversible but leads to an irreversible salt-sensitive hypertension over time [37]. Thus, SUA identification is important for risk assessment and treatment of such patients with a BMI < 21.0 kg.

An important observation was that UA may function as an antioxidant, and possibly one of the most important antioxidants in plasma. Increased SUA in subjects with CVD might therefore reflect a compensatory mechanism to counter the oxidative stress that occurs in these conditions [38]. In our study, SUA was negatively associated with hypertension in participants with a BMI ≥21.0 kg/m^2^. The beneficial antioxidant actions of SUA may partially counter its potential detrimental effects. It is interesting that almost all studies examining the relation of SUA levels with CVD events show a J-shaped curve with the nadir of risk in the second quartile [39, 40].

Some limitations of this study must be considered. First, our cross-sectional study design does not eliminate potential causal relationships between BMI, SUA, and blood pressure status. Second, the prevalence of blood pressure categories is based on single blood pressure measurement. Third, confounding factors are based on a single assessment of blood, which may introduce a misclassification bias. Fourth, we could not eliminate the possible effects of underlying diseases and medications for diabetes and dyslipidemia on the present findings. We could not rule out one-time prehypertension and white-coat prehypertension. Finally, in this study, the demographics and referral source may limit the generalization of the results.

## Conclusion

The present study showed that BMI may modify the association between SUA, SBP, and DBP. The underlying mechanism seems to be independent from traditional cardiovascular risk factors such as age, BMI, alcohol consumption, smoking status, history of CVD, lipids, diabetes, renal function, and liver function. For community-dwelling healthy persons, prospective population-based studies are needed to investigate the mechanisms underlying this association to determine whether intervention, such as effective lifestyle modifications that decrease BMI and SUA, in adult populations will decrease risks.
